# The Fruit Season

**Published:** 1857-07

**Authors:** 


### THE FRUIT SEASON.

The berries, the peaches, the apples, and the plums—not only of these, but of all others, eat freely, as often as you can get them. There are only two restrictions.

They should not be eaten later than dinner time.

They should be eaten while fresh, ripe, perfect, and in their natural raw state, without milk, cream, sugar, spices, water, or any other liquid, within an hour afterwards.

Fruits are known to be cooling and healthful; the reason is, their acidity, like that of some other articles, stimulates the separation of bile from the blood, this causes an "open" condition of the system, the attendant of high health, an active body, and a joyous heart. Hence, if that acidity is corrected by sweets of any kind, in such proportion they fail of their natural good effects.

